# Supplementary material for: Rejection-based choices discourage people from opting out of voting
Source: Nat Commun. 2026 Jan 28;17:1768. doi: 10.1038/s41467-026-68472-7 (PMC12916904; doi:10.1038/s41467-026-68472-7)
Supplement: Supplementary file 1 — Supplementary Information [file 41467_2026_68472_MOESM1_ESM.pdf]

Supplementary Information

"Rejection-based choices discourage people from opting out of voting," Yi-Hsin Su and Amitai Shenhav

Supplementary Text

1. Negative campaigning

To compare the effect of negative campaigning with that of rejection-based voting, we used our voting agent model (Study 2) to simulate voters moderately preferring Candidate A over Candidate B by 1 point (desirability 4 versus 3). We then assumed that negative campaigning on Candidate B could ideally decrease voters' preferences by 2 points (desirability 4 versus 1). We show (Supplementary Table 20) that even though the actual votes for Candidate A versus Candidate B increased (from 9% to 16%), it was because voters were less likely to vote for Candidate B (from 8% to 1%), but not more votes would be cast for Candidate A. In contrast, rejection-based voting considerably increased actual votes for Candidate A (from 18% to 59%), leading to overall higher actual votes for A versus B (from 9% to 34%).

2. Controlling for variability in demographics

All four studies employed random assignment to conditions (selection-based and rejection-based voting) to ensure that participant samples were matched for demographic and other individual-level variables. To confirm that this randomization was successful, we tested for differences in demographic variables across conditions. As expected, we found that almost all of the demographic variables collected were similarly distributed across the two groups in all four studies ($p > 0.077$; Supplementary Tables 1, 2, 3, and 4). The only exceptions to this were differences in the mother's average education level in Study 1 ($p = 0.016$) and self-reported sex assigned at birth in Study 2 ($p = 0.042$). While these effects on their own would not withstand correction for multiple comparisons across all demographic variables, as an additional conservative step, we performed two additional generalized mixed-effect regression models predicting opt-out behavior based on experimentally manipulated variables, while controlling for these two demographic variables in Studies 1 and 2, respectively (Supplementary Tables 21 and 22). We did not find the effects of these demographic variables on opt-out decisions ($p > 0.339$), and accordingly, all of our main findings remained qualitatively unchanged when controlling for these.

We performed two additional generalized mixed-effect regressions to explicitly test whether any demographic variables we collected modulated our key effect of interest (the interaction between overall desirability and choice goal conditions on the probability of out-out) in Studies 1 and 2. We included sex, age, political affiliation, race, participant's own education level, maternal education level, and parental education level in these regressions (Supplementary Table 23). We found that our framing effect holds when controlling for all these demographic variables. Moreover, we did not find consistent evidence that any of the demographic variables moderated the interaction effect (moderation by age only significant in Study 2, $p = 0.040$, but not in Study 1, $p = 0.318$; all other p's > 0.065, Supplementary Table 23).

Supplementary Figures

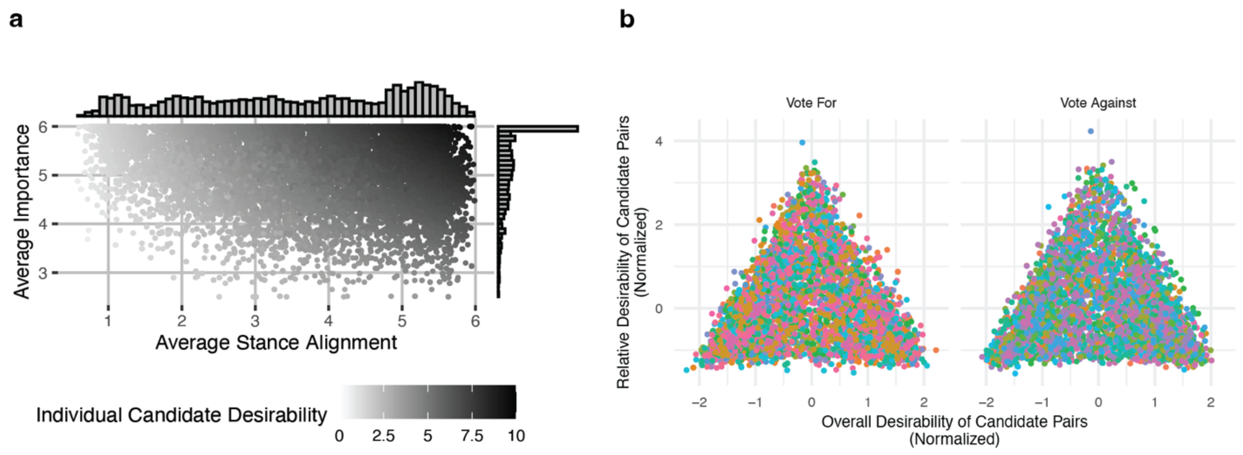

Supplementary Figure 1. Systematic Manipulations of Desirability of Candidates and Ballots. a. We synthesized a wide array of candidates who each held stances on two out of thirteen issues that had been rated by a given participant (see Figure 1a). We estimated the desirability of each candidate (white-black gradient) based on how aligned the candidates' stances were with the participants' stances on those issues (x-axis, shown as averages for visualization), weighed by how important those issues were to the participant (y-axis). The scatterplot shows desirability for all candidates shown across all participants in Study 1 (*N* = 91). **b.** We paired candidates across ballots to systematically vary their overall desirability (how desirable the two candidates were on average; x-axis) and relative desirability (how one candidate was more desirable than the other; y-axis). Scatterplots show the overall and relative desirability of all ballots shown across all participants, with each color reflecting a different participant (Left: Selection, *N* = 44; Right: Rejection, *N* = 47), demonstrating that these distributions highly overlapped as intended.

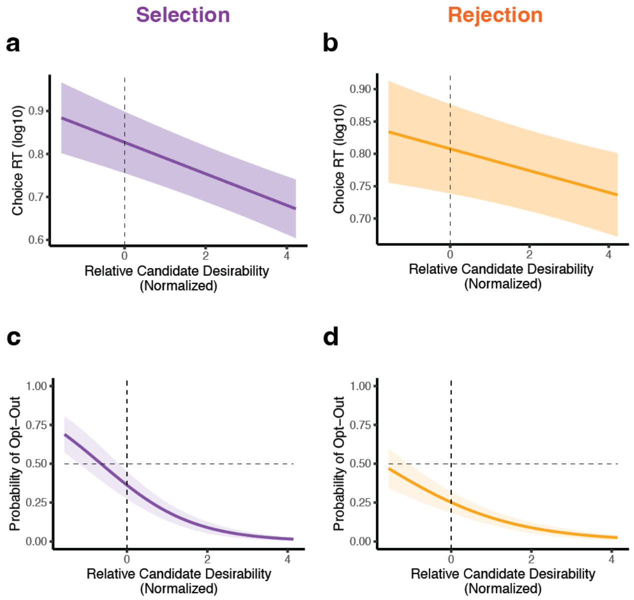

Supplementary Figure 2. Participants were faster to choose between the candidates and less likely to opt out as the difference in desirability between the candidates increased (Study 1). Participants (Select condition: *N* = 44; Reject condition: *N* = 47) were faster to select the better candidate (**a.**) or reject the worse candidate (**b.**), and less likely to be out of voting in both cases (**c.**-**d.**) when one of the candidates was much more desirable than the other (i.e., higher relative desirability). Critically, this effect was independent of the overall desirability in each of these cases, and it was covaried out in relevant analyses (see Supplementary Figure 3). Shaded error bars show 95% confidence intervals. These patterns were replicated in Study 2 (Supplementary Tables 10, 12, 15, and 16).

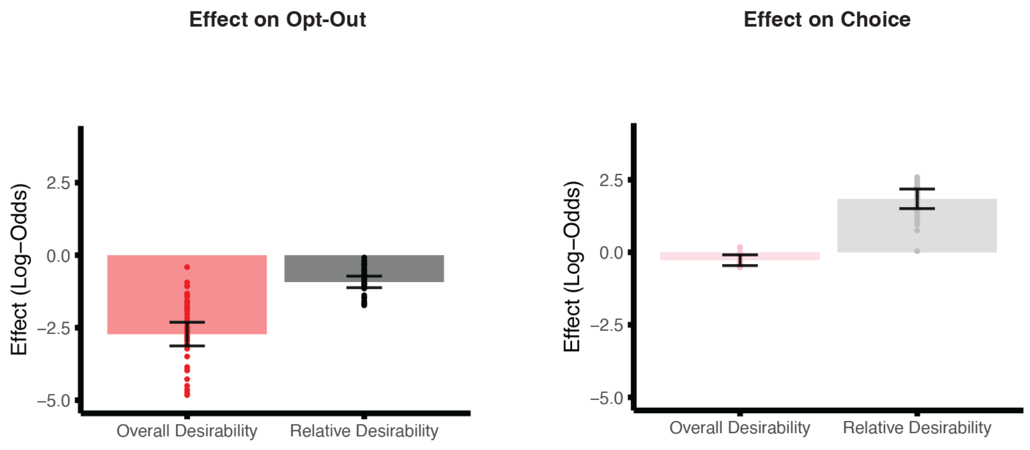

Supplementary Figure 3. Double dissociation in the influence of overall vs. relative desirability on decisions regarding whether to opt out vs. which candidate to select (Study 1). Left: The likelihood of a participant opting out of voting on a given ballot in the Select condition (higher values on the y-axis; Select participants N = 44) was primarily determined by the overall (un-)desirability of candidates and, to a lesser extent, by their relative desirability. **Right:** When participants chose to vote, the likelihood that a participant chose the more desirable candidate (higher values on the y-axis) was primarily determined by the relative desirability of the two candidates and not by overall desirability. This plot was generated from a model of candidate choice accuracy (whether the more desirable candidate is chosen; Supplementary Table 24) using unsigned relative desirability to be compared with the model of opt-out decisions (**Left**, Supplementary Table 15). These patterns were replicated in Study 2 (Supplementary Tables 15 and 24). Colored bars show magnitudes of fixed effects. Error bars show 95% confidence intervals. Data points show random slopes for each participant.

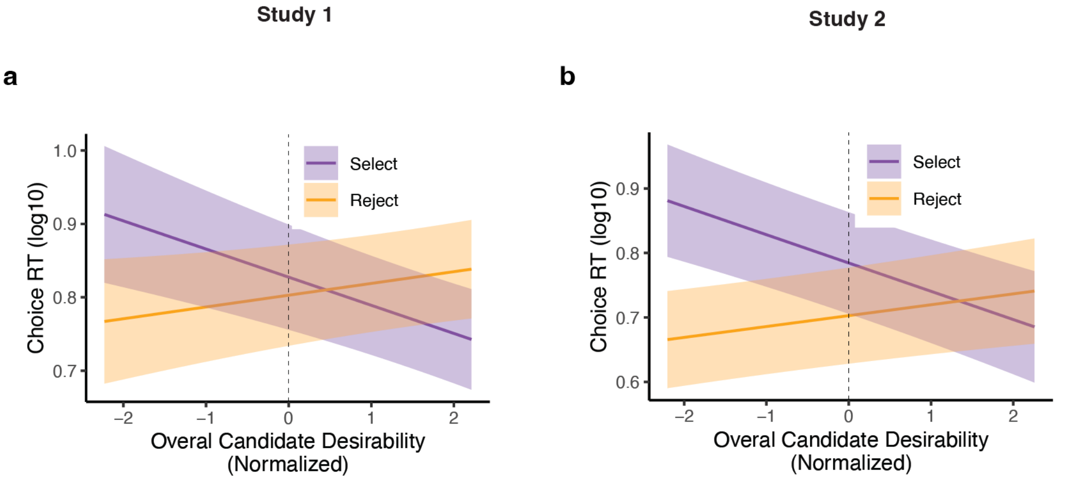

Supplementary Figure 4. Candidate choices were the fastest when selecting the better of two good candidates or rejecting the lesser of two bad candidates. Across Studies 1 (**a.**) and 2 (**b.**), response times (choice RTs) for candidate choices were negatively correlated with overall candidate desirability for the Select condition (purple lines; *N* = 44 for Study 1 and 39 for Study 2) and positively correlated with overall candidate desirability in the Reject condition (orange lines; *N* = 47 for Study 1 and 43 for Study 2). Shaded error bars show 95% confidence intervals.

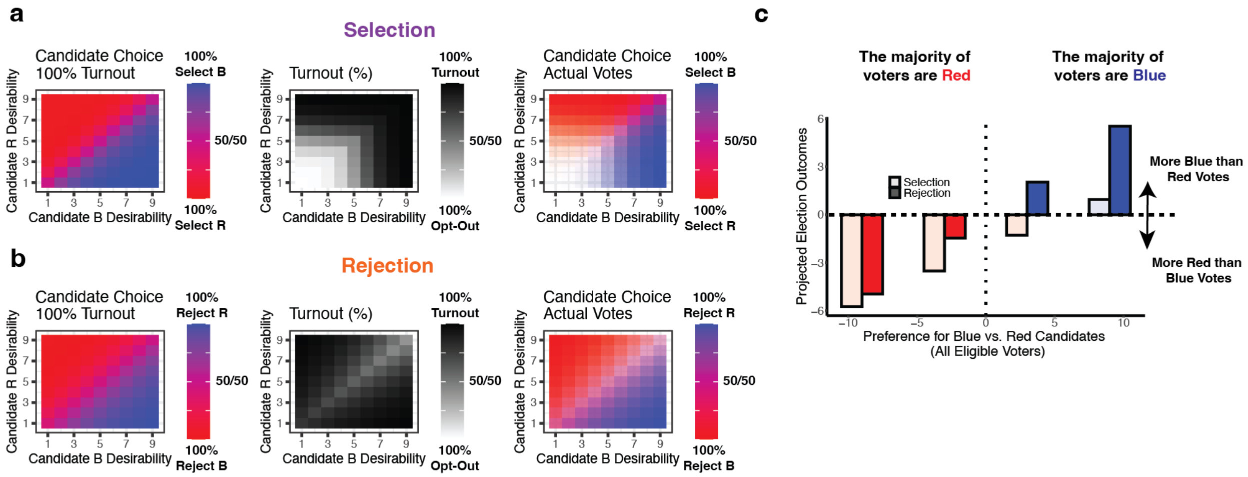

Supplementary Figure 5. Simulations of selection- and rejection-based elections based on choice data from Study 1. Here we show the results of the same simulations as shown in Figure 4 (which were based on Study 2) using data from Study 1 participants (*N* = 91). All findings replicate when using this alternate dataset (compare Panels **a-c** with Figs. 4a, 4b, and 4d).

Supplementary Tables

Supplementary Table 1. Demographics of Study 1 (Continuous Data: Brunner-Munzel (BM) tests, two-sided, df: degree of freedom; Count Data: Fisher's exact tests, two-sided, statistics shown for 2x2 tables)

Demographics	Selection (N = 44)	Rejection (N = 47)	P-value (statistics)
Sex	F/M = 19/25	F/M = 28/19	p = 0.144 (Odds = 1.92, CI = [0.78, 4.85])
Age	Mean = 37.0 (SD = 9.31)	Mean = 37.3 (SD = 9.79)	p = 0.897 (BM = 0.13, df = 88.8)
Party Affiliation	Mean = -0.63 (SD = 2.12) (N = 43)	Mean = -0.80 (SD = 1.82) (N = 46)	p = 0.854 (BM = -0.18, df = 79.3)
Race	Asian = 7<br>Black/African = 9<br>Caucasian = 20<br>Hispanic/Latinx = 1<br>Native American = 0<br>Mixed = 6<br>Other = 1	Asian = 4<br>Black/African = 8<br>Caucasian = 23<br>Hispanic/Latinx = 4<br>Native American = 1<br>Mixed = 7<br>Other = 0	p = 0.627
Hispanic/Latinx	Yes/No = 2/42	Yes/No = 8/39	p = 0.092 (Odds = 0.24, CI = [0.02, 1.28])
Education (years)	Mean = 15.8 (SD = 2.12)	Mean = 14.9 (SD = 2.81) (N = 46)	p = 0.079 (BM = -1.78, df = 76.6)
Education: Mother[1]	Mean = -0.17 (SD = 1.76) (N = 43)	Mean = 0.70 (SD = 1.51) (N = 46)	**p = 0.016 (BM = 2.46, df = 81.0)**
Education: Father[1]	Mean = -0.20 (SD = 1.64) (N = 43)	Mean = 0.39 (SD = 1.71) (N = 45)	p = 0.126 (BM = 1.55, df = 85.7)

[1] Response options included 6 education levels (see https://osf.io/djy4h), which were coded as -2.5, -1.5, -0.5, 0.5, 1.5, and 2.5.

Supplementary Table 2. Demographics of Study 2 (Continuous Data: Brunner-Munzel (BM) tests, two-sided, df: degree of freedom; Count Data: Fisher's exact tests, two-sided, statistics shown for 2x2 tables)

Demographics	Selection (N = 39)	Rejection (N = 43)	P-value (statistics)
Sex	F/M/Prefer not to respond = 14/23/2	F/M = 25/18	**p = 0.042**
Age	Mean = 33.9 (SD = 9.30)	Mean = 33.6 (SD = 9.84)	p = 0.744 (BM = -0.33, df = 80.0)
Party Affiliation	Mean = -0.95 (SD = 1.96)	Mean = -1.42 (SD = 1.68)	p = 0.350 (BM = -0.94, df = 75.8)
Race	Asian = 2<br>Black/African = 11<br>Caucasian = 21<br>Hispanic/Latinx = 3<br>Native American = 0<br>Mixed = 2<br>Other = 0	Asian = 7<br>Black/African = 9<br>Caucasian = 18<br>Hispanic/Latinx = 3<br>Native American = 0<br>Mixed = 6<br>Other = 0	p = 0.302
Hispanic/Latinx	Yes/No = 5/34	Yes/No = 6/37	p = 1.000 (Odds = 0.91, CI = [0.20, 3.94])
Education (years)	Mean = 15.1 (SD = 2.65)	Mean = 15.1 (SD = 2.22)	p = 0.696 (BM = 0.39, df = 71.5)
Education: Mother	Mean = -0.14 (SD = 1.60)	Mean = 0.36 (SD = 1.57)	p = 0.124 (BM = 1.56, df = 79.3)
Education: Father	Mean = -0.37 (SD = 1.84)	Mean = 0.17 (SD = 1.68) (N = 42)	p = 0.153 (BM = 1.44, df = 72.3)

Supplementary Table 3. Demographics of Study 3 (Continuous Data: Brunner-Munzel (BM) tests, two-sided, df: degree of freedom; Count Data: Fisher's exact tests, two-sided, statistics shown for 2x2 tables)

Demographics	Selection (*N* = 484)	Rejection (*N* = 483)	P-value (statistics)
Sex	F/M/Prefer not to respond = 251/230/3	F/M/Prefer not to respond = 237/242/4	*p* = 0.682
Age	Mean = 35.6 (SD = 9.30) (*N* = 483)	Mean = 34.9 (SD = 9.30)	*p* = 0.268 (BM = -1.1, df = 964)
Party Affiliation	Mean = -0.18 (SD = 0.89) (*N* = 482)	Mean = -0.18 (SD = 0.90) (*N* = 482)	*p* = 0.949 (BM = 0.06, df = 960)
Race	Asian = 39<br>Black/African = 45<br>Caucasian = 307<br>Hispanic/Latinx = 35<br>Native American = 8<br>Mixed = 49<br>Pacific Islander = 0<br>Other = 1	Asian = 27<br>Black/African = 53<br>Caucasian = 322<br>Hispanic/Latinx = 24<br>Native American = 2<br>Mixed = 49<br>Pacific Islander = 1<br>Other = 5	*p* = 0.078<br>(simulated, iterations = 10^5; exact value cannot be obtained)
Hispanic/Latinx	Yes/No/Prefer not to respond = 68/414/2	Yes/No/Prefer not to respond = 48/434/1	*p* = 0.090
Education (years)[2]	Mean = 15.2 (SD = 2.26)	Mean = 15.1 (SD = 2.33) (*N* = 482)	*p* = 0.535 (BM = -0.62, df = 961)
Education: Mother	Mean = 0.16 (SD = 1.61) (*N* = 474)	Mean = 0.05 (SD = 1.61) (*N* = 478)	*p* = 0.287 (BM = -1.07, df = 950)
Education: Father	Mean = 0.12 (SD = 1.70) (*N* = 459)	Mean = 0.05 (SD = 1.65) (*N* = 463)	*p* = 0.393 (BM = -0.85, df = 916)

[2]2 participants responded "less than 9", which was coded as 8.

Supplementary Table 4. Demographics of Study 4 (Continuous Data: Brunner-Munzel (BM) tests, two-sided, df: degree of freedom; Count Data: Fisher's exact tests, two-sided, statistics shown for 2x2 tables)

Demographics	Selection (*N* = 489)	Rejection (*N* = 487)	P-value (statistics)
Sex	F/M/Prefer not to respond = 252/233/4	F/M/Prefer not to respond = 282/202/3	*p* = 0.116
Age	Mean = 34.6 (SD = 9.38)	Mean = 35.5 (SD = 10.2)	*p* = 0.250 (BM = 1.15, df = 949)
Party Affiliation	Mean = -0.22 (SD = 0.98) (*N* = 486)	Mean = -0.18 (SD = 1.00)	*p* = 0.511 (BM = 0.66, df = 968)
Race	Asian = 44<br>Black/African = 65<br>Caucasian = 302<br>Hispanic/Latinx = 24<br>Native American = 0<br>Mixed = 50<br>Pacific Islander = 2<br>Other = 2	Asian = 30<br>Black/African = 72<br>Caucasian = 309<br>Hispanic/Latinx = 28<br>Native American = 2<br>Mixed = 41<br>Pacific Islander = 0<br>Other = 5	*p* = 0.237<br>(simulated, iterations = 10^5; exact value cannot be obtained)
Education (years)[3]	Mean = 15.3 (SD = 2.48) (*N* = 488)	Mean = 15.0 (SD = 2.38)	*p* = 0.194 (BM = -1.30, df = 970)

[3]3 participants responded "less than 9", which was coded as 8.

Supplementary Table 5. Other political measures in Study 3 (Brunner-Munzel (BM) tests, two-sided, df: degree of freedom)

Measures	Selection (*N* = 484)	Rejection (*N* = 483)	P-value (statistics)
Likelihood of Voting	Mean = 1.76 (SD = 1.72)	Mean = 1.60 (SD = 1.87)	*p* = 0.359 (BM = -0.92, df = 961)
Favorability: Biden	Mean = 35.5 (SD = 30.0)	Mean = 33.4 (SD = 29.5)	*p* = 0.348 (BM = -0.94, df = 965)
Trust, Biden: Economy	Mean = -0.63 (SD = 1.97)	Mean = -0.92 (SD = 1.97) (*N* = 482)	***p* = 0.022 (BM = -2.29, df = 962)**
Trust, Biden: Foreign Policy	Mean = -0.75 (SD = 1.95)	Mean = -0.95 (SD = 1.94) (*N* = 481)	*p* = 0.110 (BM = -1.60, df = 962)
Trust, Biden: Immigration Policy	Mean = -0.81 (SD = 1.89) (*N* = 483)	Mean = -0.88 (SD = 1.92) (*N* = 482)	*p* = 0.553 (BM = -0.59, df = 962)
Trust, Biden: Health Insurance	Mean = -0.19 (SD = 1.93)	Mean = -0.26 (SD = 1.87) (*N* = 481)	*p* = 0.560 (BM = -0.58, df = 961)
Trust, Biden: Gun Buying	Mean = -0.46 (SD = 1.86) (*N* = 483)	Mean = -0.65 (SD = 1.92) (*N* = 480)	*p* = 0.114 (BM = -1.58, df = 959)
Trust, Biden: Abortion	Mean = 0.30 (SD = 2.02) (*N* = 475)	Mean = 0.12 (SD = 2.07) (*N* = 479)	*p* = 0.222 (BM = -1.22, df = 952)
Favorability: Trump	Mean = 24.4 (SD = 30.8)	Mean = 22.2 (SD = 29.1)	*p* = 0.667 (BM = -0.43, df = 956)
Trust, Trump: Economy	Mean = -0.12 (SD = 2.18)	Mean = -0.30 (SD = 2.17) (*N* = 482)	*p* = 0.214 (BM = -1.24, df = 964)
Trust, Trump: Foreign Policy	Mean = -0.85 (SD = 2.15)	Mean = -0.96 (SD = 2.15) (*N* = 482)	*p* = 0.416 (BM = -0.81, df = 964)
Trust, Trump: Immigration Policy	Mean = -0.62 (SD = 2.37)	Mean = -0.70 (SD = 2.35)	*p* = 0.630 (BM = -0.48, df = 965)
Trust, Trump: Health Insurance	Mean = -1.24 (SD = 1.79) (*N* = 483)	Mean = -1.38 (SD = 1.75) (*N* = 482)	*p* = 0.198 (BM = -1.29, df = 962)
Trust, Trump: Gun Buying	Mean = -0.77 (SD = 2.08) (*N* = 483)	Mean = -0.72 (SD = 2.13) (*N* = 479)	*p* = 0.859 (BM = 0.18, df = 955)
Trust, Trump: Abortion	Mean = -1.40 (SD = 1.84) (*N* = 479)	Mean = -1.46 (SD = 1.88) (*N* = 479)	*p* = 0.420 (BM = -0.81, df = 954)

Supplementary Table 6. Other political measures in Study 4 (Brunner-Munzel (BM) tests, two-sided, df: degree of freedom)

Measures	Selection (*N* = 489)	Rejection (*N* = 487)	P-value (statistics)
Likelihood of Voting	Mean = 1.84 (SD = 1.70)	Mean = 2.06 (SD = 1.56)	***p* = 0.019 (BM = 2.36, df = 973)**
Favorability: Harris	Mean = 50.3 (SD = 33.8)	Mean = 47.2 (SD = 36.1)	*p* = 0.162 (BM = -1.40, df = 948)
Favorability: Trump	Mean = 30.2 (SD = 32.6)	Mean = 28.0 (SD = 32.8)	*p* = 0.226 (BM = -1.22, df = 974)

Supplementary Table 7. List of political issue questions used in Studies 1 and 2. We asked two questions for each issue: "Indicate your stance on [issue name (text)] using the sliding scale below the question. -3 indicates [Description for "-3" (text)] and 3 indicates [Description for "3" (text)]"; "How important is this issue to you (from -3: Not important at all to 3: Very important)?"

**issue name (text)**	**Description for "-3" (text)**	**Description for "3" (text)**
economy	government involvement in economy should definitely decrease (closer to a free market) than it is now	government involvement in economy should definitely increase (closer to a big government) than it is now
taxation	taxes on the wealthy should definitely decrease	taxes on the wealthy should definitely increase.
foreign policy	the U.S. should be less involved abroad than it is now	the U.S. should be more involved abroad than it is now.
health care	government insurance plans should dominate	private insurance plans should dominate
immigration	government should make it harder (more restrictions) for immigrants to come live in the States	government should make it easier (less restrictions) for immigrants to come live in the States.
gun policy	the government should make it harder than it is now to buy a gun	the government should make it easier than it is now to buy a gun
social security	spending on social security should definitely decrease	spending on social security should definitely increase
education	spending on education should definitely decrease	spending on education should definitely increase
Supreme Court appointment	should definitely appoint liberal judges	should definitely appoint conservative judges
police forces	the federal government should definitely decrease regulations on local police forces	the federal government should definitely increase regulations on local police forces
environment	government should not regulate business to protect the environment	government should regulate business to protect the environment
abortion	legally allow abortions in all circumstances	outlaw abortions in all circumstances
LGBTQ+	definitely support protecting members of the LGBTQ+ community from discrimination	definitely oppose protecting members of the LGBTQ+ community from discrimination

Supplementary Table 8. List of political issue scales used in Studies 1 and 2. Below each question, participants saw a sliding scale on which they indicated their answers. Below are those texts on the scales and the summary statistics of stance and importance ratings for each issue. We combined the ratings from Studies 1 and 2 participants (*N* = 173) who performed the voting tasks in March 2024. (Wilcoxon signed rank tests, two-sided, null hypothesis: median = 0)

**issue name (scale)**	**Description for "-3" (scale)**	**Description for "3" (scale)**	**Stance Ratings: Mean (SD, p-value, statistic)**	**Importance Ratings: Mean (SD, p-value, statistic)**
Government Involvement in Economy	Decrease	Increase	0.73 (1.53, $p < 0.001$, $V = 11059$)	1.08 (1.29, $p < 0.001$, $V = 13076$)
Taxation on the Wealthy	Decrease	Increase	2.00 (1.32, $p < 0.001$, $V = 14321$)	1.38 (1.42, $p < 0.001$, $V = 13158$)
US Foreign Involvement	Less	More	-0.63 (1.53, $p < 0.001$, $V = 3918$)	0.58 (1.52, $p < 0.001$, $V = 10596$)
Health Insurance	Government	Private	-1.16 (1.78, $p < 0.001$, $V = 2632$)	1.70 (1.36, $p < 0.001$, $V = 14002$)
Immigration to the US	Harder	Easier	0.13 (1.79, $p = 0.438$, $V = 7588$)	1.03 (1.42, $p < 0.001$, $V = 12449$)
Gun Buying	Harder	Easier	-1.83 (1.55, $p < 0.001$, $V = 1051$)	1.61 (1.47, $p < 0.001$, $V = 13774$)
Spending on Social Security	Decrease	Increase	1.30 (1.36, $p < 0.001$, $V = 13406$)	1.05 (1.39, $p < 0.001$, $V = 12644$)
Spending on Education	Decrease	Increase	1.93 (1.27, $p < 0.001$, $V = 14306$)	1.66 (1.29, $p < 0.001$, $V = 14240$)
Supreme Court Judges	Liberal	Conservative	-0.94 (1.78, $p < 0.001$, $V = 3083$)	1.49 (1.41, $p < 0.001$, $V = 13364$)
Government Regulating Local Police Forces	Decrease	Increase	1.16 (1.47, $p < 0.001$, $V = 12885$)	1.06 (1.32, $p < 0.001$, $V = 12836$)
Regulating Business Impact on the Environment	No	Yes	1.75 (1.32, $p < 0.001$, $V = 14293$)	1.44 (1.31, $p < 0.001$, $V = 13818$)
Abortion	Legal	Illegal	-1.62 (2.00, $p < 0.001$, $V = 2068$)	1.83 (1.43, $p < 0.001$, $V = 14124$)
Protecting LGBTQ+ from Discrimination	Support	Oppose	-1.67 (1.88, $p < 0.001$, $V = 2004$)	1.02 (2.00, $p < 0.001$, $V = 11226$)

Supplementary Table 9. Fix-effect coefficients: models of candidate choices in the Select groups (Generalized linear mixed-effect models; tests: two-sided, no adjustments for multiple comparisons)

Left is Chosen (Left: 1; Right: 0)	Study 1 (*N* = 44)		Study 2 (*N* = 39)	
	Log-Odds (z-value)	CI (p-value)	Log-Odds (z-value)	CI (p-value)
Intercept	0.09 (0.81)	[-0.12, 0.29] $p = 0.421$	0.06 (0.65)	[-0.12, 0.24] $p = 0.518$
Relative Desirability (Left vs. Right)	**2.75 (13.9)**	**[2.36, 3.14]** $\mathbf{p < 0.001}$	**2.22 (13.3)**	**[1.90, 2.55]** $\mathbf{p < 0.001}$
Overall Desirability	0.05 (0.66)	[-0.11, 0.22] $p = 0.511$	-0.09 (-1.25)	[-0.24, 0.05] $p = 0.210$
Trial Order	-0.02 (-0.16)	[-0.20, -0.17] $p = 0.870$	0.17 (1.94)	[-0.00, 0.35] $p = 0.052$
Relative Desirability (Left vs. Right) X Trial Order	0.04 (0.35)	[-0.20, 0.29] $p = 0.725$	0.07 (0.72)	[-0.13, 0.28] $p = 0.473$
Overall Desirability X Trial Order	0.03 (0.37)	[-0.13, 0.18] $p = 0.712$	-0.13 (-1.76)	[-0.28, 0.01] $p = 0.078$

Supplementary Table 10. Fix-effect coefficients: models of candidate choice RTs in the Select groups (Linear mixed-effect models, tests: two-sided, no adjustments for multiple comparisons; df: degree of freedom)

Log10 (candidate choice RT)	Study 1 (*N* = 44)		Study 2 (*N* = 39)	
	Beta (*t*, df)	CI (p-value)	Beta (*t*, df)	CI (p-value)
Intercept	**0.83 (26.4, 44.7)**	**[0.77, 0.90] *p* < 0.001**	**0.79 (28.6, 37.5)**	**[0.73, 0.85] *p* < 0.001**
Relative Desirability	**-0.04 (-5.61, 45.0)**	**[-0.05, -0.02] *p* < 0.001**	**-0.03 (-4.42, 33.1)**	**[-0.04, -0.02] *p* < 0.001**
Overall Desirability	**-0.04 (-4.47, 38.0)**	**[-0.06, -0.02] *p* < 0.001**	**-0.05 (-5.56, 28.9)**	**[-0.06, -0.03] *p* < 0.001**
Trial Order	**-0.04 (-4.67, 45.8)**	**[-0.06, -0.02] *p* < 0.001**	**-0.03 (-4.02, 33.0)**	**[-0.05, -0.02] *p* < 0.001**
Relative Desirability X Trial Order	-0.00 **(-0.80, 182)**	[-0.01, 0.01] *p* = 0.425	-0.00 **(-0.74, 425)**	[-0.01, 0.01] *p* = 0.463
Overall Desirability X Trial Order	-0.00 **(-0.69, 60.8)**	[-0.02, 0.01] *p* = 0.494	**-0.02 (-2.46, 39.3)**	**[-0.03, -0.00] *p* = 0.019**

Supplementary Table 11. Fix-effect coefficients: models of candidate choices in the Reject groups (Generalized linear mixed-effect models, tests: two-sided, no adjustments for multiple comparisons)

Left is Chosen (Left: 1; Right: 0)	Study 1 (*N* = 47)		Study 2 (*N* = 43)	
	Log-Odds (z-value)	CI (p-value)	Log-Odds (z-value)	CI (p-value)
Intercept	0.02 (0.24)	[-0.11, 0.14] *p* = 0.807	0.01 (0.17)	[-0.10, 0.12] *p* = 0.868
Relative Desirability (Left vs. Right)	**-1.67 (-11.1)**	**[-1.97, -1.38] *p* < 0.001**	**-1.85 (-12.5)**	**[-2.14, -1.56] *p* < 0.001**
Overall Desirability	-0.06 (-1.08)	[-0.17, 0.05] *p* = 0.281	-0.03 (-0.57)	[-0.12, 0.07] *p* = 0.566
Trial Order	**-0.12 (-2.03)**	**[-0.23, -0.00] *p* = 0.042**	0.06 (1.40)	[-0.03, 0.16] *p* = 0.162
Relative Desirability (Left vs. Right) X Trial Order	**-0.17 (-2.12)**	**[-0.32, -0.01] *p* = 0.034**	0.04 (0.70)	[-0.07, 0.15] *p* = 0.485
Overall Desirability X Trial Order	0.02 (0.32)	[-0.08, 0.12] *p* = 0.749	0.04 (0.92)	[-0.05, 0.13] *p* = 0.359

Supplementary Table 12. Fix-effect coefficients: models of candidate choice RTs in the Reject groups (Linear mixed-effect models, tests: two-sided, no adjustments for multiple comparisons; df: degree of freedom)

Log10 (candidate choice RT)	Study 1 (*N* = 47)		Study 2 (*N* = 43)	
	Beta (*t*, df)	CI (p-value)	Beta (*t*, df)	CI (p-value)
Intercept	**0.80 (20.3, 46.0)**	**[0.72, 0.88] *p* < 0.001**	**0.70 (15.0, 42.0)**	**[0.61, 0.80] *p* < 0.001**
Relative Desirability	**-0.02 (-2.76, 49.1)**	**[-0.03, -0.00] *p* = 0.008**	**-0.02 (-2.96, 43.4)**	**[-0.03, -0.01] *p* = 0.005**
Overall Desirability	**0.02 (2.06, 39.1)**	**[0.00, 0.03] *p* = 0.046**	**0.02 (2.80, 39.5)**	**[0.00, 0.03] *p* = 0.008**
Trial Order	**-0.06 (-5.44, 44.8)**	**[-0.08, -0.04] *p* < 0.001**	**-0.05 (-4.84, 41.5)**	**[-0.07, -0.03] *p* < 0.001**
Relative Desirability X Trial Order	0.01 (1.89, 70.3)	[-0.00, 0.02] *p* = 0.062	0.00 (0.19, 380)	[-0.01, 0.01] *p* = 0.851
Overall Desirability X Trial Order	0.00 (0.05, 51.4)	[-0.01, 0.01] *p* = 0.958	-0.01 (-1.13, 71.7)	[-0.02, 0.01] *p* = 0.261

Supplementary Table 13. Fix-effect coefficients: models of candidate choices in both groups (Generalized linear mixed-effect models, tests: two-sided, no adjustments for multiple comparisons)

Left is Chosen (Left: 1; Right: 0)	Study 1 (*N* = 91)		Study 2 (*N* = 82)	
	Log-Odds (z-value)	CI (p-value)	Log-Odds (z-value)	CI (p-value)
Intercept	0.06 (1.10)	[-0.05, 0.17] *p* = 0.269	0.01 (0.22)	[-0.09, 0.11] *p* = 0.825
Task (-1: Select; 1: Reject)	-0.03 (-0.53)	[-0.15, 0.08] *p* = 0.597	-0.02 (-0.43)	[-0.13, 0.08] *p* = 0.668
Relative Desirability (Left vs. Right)	**0.50 (4.31)**	**[0.27, 0.73] *p* < 0.001**	0.19 (1.70)	[-0.03, 0.40] *p* = 0.089
Overall Desirability	0.01 (0.19)	[-0.08, 0.10] *p* = 0.852	-0.07 (-1.60)	[-0.15, 0.02] *p* = 0.109
Trial Order	-0.05 (-0.97)	[-0.14, 0.05] *p* = 0.334	0.08 (1.74)	[-0.01, 0.16] *p* = 0.082
Relative Desirability (Left vs. Right) X Trial Order	0.02 (0.42)	[-0.09, 0.14] *p* = 0.673	0.06 (1.09)	[-0.05, 0.16] *p* = 0.275
Overall Desirability X Trial Order	0.01 (0.12)	[-0.08, 0.09] *p* = 0.906	-0.02 (-0.58)	[-0.10, 0.06] *p* = 0.565
Task (-1: Select; 1: Reject) X Relative Desirability (Left vs. Right)	**-2.19 (-18.0)**	**[-2.43, -1.95] *p* < 0.001**	**-2.05 (-18.1)**	**[-2.27, -1.82] *p* < 0.001**
Task (-1: Select; 1: Reject) X Overall Desirability	-0.05 (-1.12)	[-0.15, 0.04] *p* = 0.264	0.02 (0.45)	[-0.07, 0.10] *p* = 0.653
Task (-1: Select; 1: Reject) X Trial Order	-0.07 (-1.34)	[-0.17, 0.03] *p* = 0.180	-0.03 (-0.63)	[-0.12, 0.06] *p* = 0.526
Task (-1: Select; 1: Reject) X Relative Desirability (Left vs. Right) X Trial Order	-0.13 (-1.88)	[-0.27, 0.01] *p* = 0.060	0.01 (0.22)	[-0.11, 0.14] *p* = 0.828
Task (-1: Select; 1: Reject) X Overall Desirability X Trial Order	0.00 (0.08)	[-0.08, 0.09] *p* = 0.939	0.06 (1.47)	[-0.02, 0.14] *p* = 0.142

Supplementary Table 14. Fix-effect coefficients: models of candidate choice RTs in both groups (Linear mixed-effect models, two-sided, no adjustments for multiple comparisons; df: degree of freedom)

Log10 (candidate choice RT)	Study 1 (N = 91)		Study 2 (N = 82)	
	Beta (*t*, df)	CI (p-value)	Beta (*t*, df)	CI (p-value)
Intercept	**0.82 (31.9, 90.6)**	**[0.77, 0.87] *p* < 0.001**	**0.75 (26.8, 81.2)**	**[0.69, 0.80] *p* < 0.001**
Task (-1: Select; 1: Reject)	-0.01 (-0.54, 90.6)	[-0.06, 0.04] *p* = 0.587	-0.04 (-1.51, 81.2)	[-0.10, 0.01] *p* = 0.136
Relative Desirability	**-0.03 (-0.61, 97.7)**	**[-0.04, -0.02] *p* < .001**	**-0.02 (-5.47, 92.2)**	**[-0.03, -0.02] *p* < 0.001**
Overall Desirability	-0.01 (-1.90, 83.0)	[-0.02, 0.00] *p* = 0.061	**-0.01 (-2.77, 104)**	**[-0.02, -0.00] *p* = 0.007**
Trial Order	**-0.05 (-6.87, 102)**	**[-0.06, -0.03] *p* < .001**	**-0.04 (-5.91, 98.3)**	**[-0.05, -0.03] *p* < 0.001**
Relative Desirability X Trial Order	0.00 (0.67, 201)	[-0.00, 0.01] *p* = 0.504	-0.00 (-0.66, 2276)	[-0.01, 0.00] *p* = 0.512
Overall Desirability X Trial Order	-0.00 (-0.54, 134)	[-0.01, 0.01] *p* = 0.589	**-0.01 (-2.76, 333)**	**[-0.02, -0.00] *p* = 0.006**
Task (-1: Select; 1: Reject) X Relative Desirability	**0.01 (2.30, 97.7)**	**[0.00, 0.02] *p* = 0.024**	0.01 (1.46, 92.2)	[-0.00, 0.01] *p* = 0.147
Task (-1: Select; 1: Reject) Overall Desirability	**0.03 (4.62, 83.0)**	**[0.02, 0.04] *p* < 0.001**	**0.03 (6.07, 104)**	**[0.02, 0.04] *p* < 0.001**
Task (-1: Select; 1: Reject) Trial Order	-0.01(-1.24, 102)	[-0.02, 0.01] *p* = 0.217	-0.01 (-1.55, 98.3)	[-0.02, 0.00] *p* = 0.123
Task (-1: Select; 1: Reject) X Relative Desirability (Left vs. Right) X Trial Order	**0.01 (2.04, 201)**	**[0.00, 0.01] *p* = 0.043**	0.00 (0.77, 2276)	[-0.00, 0.01] *p* = 0.443
Task (-1: Select; 1: Reject) X Overall Desirability X Trial Order	0.00 (0.79, 134)	[-0.01, 0.01] *p* = 0.432	0.01 (1.29, 333)	[-0.00, 0.02] *p* = 0.198

Supplementary Table 15. Fix-effect coefficients: models of opt-out decisions in the Select groups (Generalized linear mixed-effect models, tests: two-sided, no adjustments for multiple comparisons)

Opt-Out (Opt-Out: 1; Opt-to-Vote: 0)	Study 1 (*N* = 44)		Study 2 (*N* = 39)	
	Log-Odds (z-value)	CI (p-value)	Log-Odds (z-value)	CI (p-value)
Intercept	**-0.53 (-2.41)**	**[-0.97, -0.10] *p* = 0.016**	**-0.85 (-4.31)**	**[-1.23, -0.46] *p* < 0.001**
Relative Desirability	**-0.92 (-9.05)**	**[-1.12, -0.72] *p* < 0.001**	**-0.88 (-7.67)**	**[-1.10, -0.65] *p* < 0.001**
Overall Desirability	**-2.72 (-13.1)**	**[-3.13, -2.31] *p* < 0.001**	**-2.89 (-13.0)**	**[-3.33, -2.46] *p* < 0.001**
Trial Order	0.12 (1.48)	[-0.04, 0.28] *p* = 0.139	**0.25 (3.13)**	**[0.09, 0.41] *p* = 0.002**
Relative Desirability X Trial Order	-0.03 (-0.45)	[-0.17, 0.11] *p* = 0.655	-0.00 (-0.04)	[-0.16, 0.15] *p* = 0.967
Overall Desirability X Trial Order	-0.11 (-1.04)	[-0.32, 0.10] *p* = 0.298	-0.07 (-0.54)	[-0.31, 0.18] *p* = 0.588

Supplementary Table 16. Fix-effect coefficients: models of opt-out decisions in the Reject groups (Generalized linear mixed-effect models, tests: two-sided, no adjustments for multiple comparisons)

Opt-Out (Opt-Out: 1; Opt-to-Vote: 0)	Study 1 (*N* = 47)		Study 2 (*N* = 43)	
	Log-Odds (z-value)	CI (p-value)	Log-Odds (z-value)	CI (p-value)
Intercept	**-1.10 (-6.99)**	**[-1.41, -0.79] *p* < 0.001**	**-1.83 (-9.77)**	**[-2.20, -1.46] *p* < 0.001**
Relative Desirability	**-0.63 (-6.99)**	**[-0.81, -0.46] *p* < 0.001**	**-0.63 (-7.16)**	**[-0.80, -0.46] *p* < 0.001**
Overall Desirability	**0.71 (2.65)**	**[0.19, 1.24] *p* = 0.008**	0.16 (0.72)	[-0.28, 0.60] *p* = 0.469
Trial Order	0.07 (0.71)	[-0.12, 0.25] *p* = 0.477	0.05 (0.54)	[-0.12, 0.22] *p* = 0.590
Relative Desirability X Trial Order	0.01 (0.20)	[-0.11, 0.13] *p* = 0.838	0.06 (0.84)	[-0.07, 0.19] *p* = 0.403
Overall Desirability X Trial Order	**0.19 (2.08)**	**[0.01, 0.36] *p* = 0.037**	0.10 (1.23)	[-0.06, 0.25] *p* = 0.217

Supplementary Table 17. Fix-effect coefficients: models of opt-out decisions in both groups (Generalized linear mixed-effect models, tests: two-sided, no adjustments for multiple comparisons)

Opt-Out (Opt-Out: 1; Opt-to-Vote: 0)	Study 1 (*N* = 91)		Study 2 (*N* = 82)	
	Log-Odds (z-value)	CI (p-value)	Log-Odds (z-value)	CI (p-value)
Intercept	**-0.83 (-6.17)**	**[-1.09, -0.56] *p* < 0.001**	**-1.37 (-10.1)**	**[-1.63, -1.10] *p* < 0.001**
Task (-1: Select; 1: Reject)	-0.26 (-1.95)	[-0.52, 0.00] *p* = 0.051	**-0.47 (-3.49)**	**[-0.73, -0.21] *p* < 0.001**
Relative Desirability	**-0.75 (-11.5)**	**[-0.87, -0.62] *p* < 0.001**	**-0.72 (-10.3)**	**[-0.85, -0.58] *p* < 0.001**
Overall Desirability	**-1.04 (-6.22)**	**[-1.37, -0.71] *p* < 0.001**	**-1.36 (-8.91)**	**[-1.66, -1.06] *p* < 0.001**
Trial Order	0.09 (1.44)	[-0.03, 0.21] *p* = 0.150	**0.15 (2.46)**	**[0.03, 0.27] *p* = 0.014**
Relative Desirability X Trial Order	-0.03 (-0.64)	[-0.11, 0.05] *p* = 0.519	0.01 (0.17)	[-0.08, 0.10] *p* = 0.865
Overall Desirability X Trial Order	-0.06 (-0.93)	[-0.18, 0.06] *p* = 0.354	-0.05 (-0.75)	[-0.18, 0.08] *p* = 0.451
Task (-1: Select; 1: Reject) X Relative Desirability	**0.13 (2.00)**	**[0.00, 0.25] *p* = 0.045**	0.07 (1.00)	[-0.06, 0.20] *p* = 0.317
Task (-1: Select; 1: Reject) X Overall Desirability	**1.71 (10.2)**	**[1.38, 2.04] *p* < 0.001**	**1.54 (10.1)**	**[1.24, 1.84] *p* < 0.001**
Task (-1: Select; 1: Reject) X Trial Order	-0.01 (-0.23)	[-0.13, 0.11] *p* = 0.819	-0.07 (-1.27)	[-0.19, 0.04] *p* = 0.205
Task (-1: Select; 1: Reject) X Relative Desirability X Trial Order	0.05 (1.22)	[-0.03, 0.12] *p* = 0.221	0.04 (0.92)	[-0.04, 0.12] *p* = 0.358
Task (-1: Select; 1: Reject) X Overall Desirability X Trial Order	**0.23 (3.63)**	**[0.11, 0.35] *p* < 0.001**	**0.14 (2.10)**	**[0.01, 0.26] *p* = 0.035**

Supplementary Table 18. Proportions of uncommitted voters in Study 3 (Two-proportion, one-tailed z tests, one-sided; df: degree of freedom)

	Preregistered Prediction: Primary (Include "Prefer not to respond")		Preregistered Prediction: Secondary (Exclude "Prefer not to respond")	
Condition	Selection	Rejection	Selection	Rejection
Committed Voters	325	387	325	387
Uncommitted Voters	168	98	159	96
Proportions	0.341	0.202	0.329	0.199
Two-proportion test (one-tailed)	chi-square = 23.1, df = 1<br>*p* <.001		chi-square = 20.3, df = 1<br>*p* <.001	

Supplementary Table 19. Proportions of uncommitted voters in Study 4 (Two-proportion, one-tailed z tests, one-sided; df: degree of freedom)

	Preregistered Prediction: Primary (Include "Prefer not to respond")		Preregistered Prediction: Secondary (Exclude "Prefer not to respond")	
Condition	Selection	Rejection	Selection	Rejection
Committed Voters	377	416	377	416
Uncommitted Voters	117	74	112	71
Proportions	0.237	0.151	0.229	0.146
Two-proportion test (one-tailed)	chi-square = 11.0, df = 1<br>*p* <.001		chi-square = 10.6, df = 1<br>*p* <.001	

Supplementary Table 20. Simulations of negative campaigning

	Desirability: Candidate A	Desirability: Candidate B	Actual Votes for A (per 1 vote)	Actual Votes for B (per 1 vote)	Goal	Actual Votes for A (vs. B, per 1 vote)
Baseline	4	3	0.176	0.082	Selection	0.094
Negative Campaigning	4	1	0.172	0.012	Selection	0.160
Rejection-Based Voting	4	3	0.587	0.243	Rejection	0.344

Supplementary Table 21. Controlling for variability in demographics: Study 1 (Generalized linear mixed-effect models, tests: two-sided, no adjustments for multiple comparisons)

Opt-Out (Opt-Out: 1; Opt-to-Vote: 0)	Study 1 (*N* = 91) (without controlling Mother Education)		Study 1 (*N* = 89) (with controlling Mother Education)	
	Log-Odds (z-value)	CI (p-value)	Log-Odds (z-value)	CI (p-value)
Intercept	**-0.83 (-6.17)**	**[-1.09, -0.56] *p* < 0.001**	**-0.85 (-6.25)**	**[-1.12, -0.59] *p* < 0.001**
Mother Education			0.11 (0.95)	[-0.11, 0.33] *p* = 0.340
Task (-1: Select; 1: Reject)	-0.26 (-1.95)	[-0.52, 0.00] *p* = 0.051	**-0.31 (-2.19)**	**[-0.58, -0.03] *p* = 0.029**
Relative Desirability	**-0.75 (-11.5)**	**[-0.87, -0.62] *p* < 0.001**	**-0.75 (-11.2)**	**[-0.88, -0.62] *p* < 0.001**
Overall Desirability	**-1.04 (-6.22)**	**[-1.37, -0.71] *p* < 0.001**	**-1.02 (-5.99)**	**[-1.35, -0.68] *p* < 0.001**
Trial Order	0.09 (1.44)	[-0.03, 0.21] *p* = 0.150	0.09 (1.49)	[-0.03, 0.22] *p* = 0.135
Relative Desirability X Trial Order	-0.03 (-0.64)	[-0.11, 0.05] *p* = 0.519	-0.04 (-0.84)	[-0.12, 0.05] *p* = 0.402
Overall Desirability X Trial Order	-0.06 (-0.93)	[-0.18, 0.06] *p* = 0.354	-0.06 (-0.90)	[-0.18, 0.07] *p* = 0.368
Task (-1: Select; 1: Reject) X Relative Desirability	**0.13 (2.00)**	**[0.00, 0.25] *p* = 0.045**	0.12 (1.85)	[-0.01, 0.25] *p* = 0.064
Task (-1: Select; 1: Reject) X Overall Desirability	**1.71 (10.2)**	**[1.38, 2.04] *p* < 0.001**	**1.74 (10.2)**	**[1.41, 2.07] *p* < .001**
Task (-1: Select; 1: Reject) X Trial Order	-0.01 (-0.23)	[-0.13, 0.11] *p* = 0.819	-0.02 (-0.39)	[-0.15, 0.10] *p* = 0.695
Task (-1: Select; 1: Reject) X Relative Desirability X Trial Order	0.05 (1.22)	[-0.03, 0.12] *p* = 0.221	0.05 (1.31)	[-0.03, 0.13] *p* = 0.191
Task (-1: Select; 1: Reject) X Overall Desirability X Trial Order	**0.23 (3.63)**	**[0.11, 0.35] *p* < 0.001**	**0.23 (3.59)**	**[0.11, 0.36] *p* < 0.001**

Supplementary Table 22. Controlling for variability in demographics: Study 2 (Generalized linear mixed-effect models, two-sided, no adjustments for multiple comparisons)

Opt-Out (Opt-Out: 1; Opt-to-Vote: 0)	Study 2 (*N* = 82) (without controlling Sex)		Study 2 (*N* = 82) (with controlling Sex)	
	Log-Odds (z-value)	CI (p-value)	Log-Odds (z-value)	CI (p-value)
Intercept	**-1.37 (-10.1)**	**[-1.63, -1.10] *p* < 0.001**	**-1.14 (-3.98)**	**[-1.70, -0.58] *p* < 0.001**
Sex: Male vs. Female			-0.05 (-0.42)	[-0.30, 0.19] *p* = 0.675
Sex: Prefer not to respond			0.25 (0.91)	[-0.29, 0.79] *p* = 0.364
Task (-1: Select; 1: Reject)	**-0.47 (-3.49)**	**[-0.73, -0.21] *p* < 0.001**	**-0.46 (-3.34)**	**[-0.73, -0.19] *p* = 0.001**
Relative Desirability	**-0.72 (-10.3)**	**[-0.85, -0.58] *p* < 0.001**	**-0.72 (-10.3)**	**[-0.85, -0.58] *p* < 0.001**
Overall Desirability	**-1.36 (-8.91)**	**[-1.66, -1.06] *p* < 0.001**	**-1.36 (-8.91)**	**[-1.67, -1.06] *p* < 0.001**
Trial Order	**0.15 (2.46)**	**[0.03, 0.27] *p* = 0.014**	**0.15 (2.47)**	**[0.03, 0.27] *p* = 0.013**
Relative Desirability X Trial Order	0.01 (0.17)	[-0.08, 0.10] *p* = 0.865	0.01 (0.16)	[-0.08, 0.10] *p* = 0.875
Overall Desirability X Trial Order	-0.05 (-0.75)	[-0.18, 0.08] *p* = 0.451	-0.05 (-0.74)	[-0.17, 0.08] *p* = 0.458
Task (-1: Select; 1: Reject) X Relative Desirability	0.07 (1.00)	[-0.06, 0.20] *p* = 0.317	0.07 (1.00)	[-0.06, 0.20] *p* = 0.315
Task (-1: Select; 1: Reject) X Overall Desirability	**1.54 (10.1)**	**[1.24, 1.84] *p* < 0.001**	**1.54 (10.1)**	**[1.24, 1.84] *p* < 0.001**
Task (-1: Select; 1: Reject) X Trial Order	-0.07 (-1.27)	[-0.19, 0.04] *p* = 0.205	-0.08 (-1.30)	[-0.19, 0.04] *p* = 0.194
Task (-1: Select; 1: Reject) X Relative Desirability X Trial Order	0.04 (0.92)	[-0.04, 0.12] *p* = 0.358	0.04 (0.92)	[-0.04, 0.12] *p* = 0.356
Task (-1: Select; 1: Reject) X Overall Desirability X Trial Order	**0.14 (2.10)**	**[0.01, 0.26] *p* = 0.035**	**0.14 (2.13)**	**[0.01, 0.26] *p* = 0.033**

Supplementary Table 23. Moderation effects on Task X Overall Desirability: Study 1 and 2 (Generalized linear mixed-effect models, tests: two-sided, no adjustments for multiple comparisons)

Opt-Out<br>(Opt-Out: 1; Opt-to-Vote: 0)	Study 1 (*N* = 85)		Study 2 (*N* = 79)	
	Log-Odds<br>(z-value)	CI (p-value)	Log-Odds<br>(z-value)	CI (p-value)
Task (-1: Select; 1: Reject)<br>X Overall Desirability	**1.79 (6.08)**	**[1.21, 2.36] *p* <.001**	**1.72 (6.43)**	**[1.20, 2.25] *p* <.001**
Moderating Variable Y<br>(Y X Task X Overall Desirability)				
Y = Sex (-1: Female; 1: Male)	0.09 (0.49)	[-0.26, 0.44] *p* = 0.621	-0.06 (-0.34)	[-0.38, 0.27] *p* = 0.731
Y = Race, Contrast 1[4]	-0.21 (-0.87)	[-0.70, 0.27] *p* = 0.387	-0.08 (-0.45)	[-0.42, 0.26] *p* = 0.649
Race, Contrast 2[4]	-0.15 (-0.78)	[-0.51, 0.22] *p* = 0.436	0.19 (0.74)	[-0.31, 0.69] *p* = 0.458
Race, Contrast 3[4]	0.09 (0.40)	[-0.35, 0.53] *p* = 0.692	0.16 (1.03)	[-0.14, 0.46] *p* = 0.301
Race, Contrast 4[4]	-0.13 (-1.29)	[-0.33, 0.07] *p* = 0.197	0.03 (0.21)	[-0.21, 0.26] *p* = 0.831
Y = Age	-0.19 (-1.00)	[-0.55, 0.18] *p* = 0.318	**-0.31 (-2.05)**	**[-0.61, -0.01] *p* = 0.040**
Y = Party Affiliation - linear	-0.15 (-0.77)	[-0.51, 0.22] *p* = 0.439	-0.25 (-1.36)	[-0.62, 0.11] *p* = 0.172
Y = Party Affiliation - quadratic	-0.14 (-0.73)	[-0.52, 0.24] *p* = 0.465	-0.03 (-0.17)	[-0.35, 0.30] *p* = 0.866
Y = Education (years)	0.28 (1.52)	[-0.08, 0.65] *p* = 0.130	0.28 (1.69)	[-0.04, 0.60] *p* = 0.091
Y = Education: Mother	-0.39 (-1.73)	[-0.84, 0.05] *p* = 0.083	0.24 (1.07)	[-0.20, 0.69] *p* = 0.286
Y = Education: Father	0.12 (0.56)	[-0.30, 0.54] *p* = 0.577	-0.42 (-1.84)	[-0.87, 0.03] *p* = 0.066

[4]Contrast Coding: 1 -> White = -1, Black/African = 1, Asian = 0, Hispanic = 0, Others = 0.
Contrast Coding: 2 -> White = -1, Black/African = -1, Asian = 2, Hispanic = 0, Others = 0.
Contrast Coding: 3 -> White = -1, Black/African = -1, Asian = -1, Hispanic = 3, Others = 0.
Contrast Coding: 4 -> White = -1, Black/African = -1, Asian = -1, Hispanic = -1, Others = 4.

Supplementary Table 24. Fix-effect coefficients: candidate choice accuracy in the Select group (Generalized linear mixed-effect models, tests: two-sided, no adjustments for multiple comparisons)

Accuracy (More desirable is chosen: 1; otherwise: 0)	Study 1 (*N* = 44)		Study 2 (*N* = 39)	
	Log-Odds<br>(z-value)	CI (p-value)	Log-Odds<br>(z-value)	CI (p-value)
Intercept	**2.33 (11.6)**	**[1,94, 2.72] *p* < 0.001**	**1.85 (11.2)**	**[1.53, 2.18] *p* < 0.001**
Relative Desirability	**1.84 (10.7)**	**[1.50, 2.18] *p* < 0.001**	**1.51 (10.7)**	**[1.23, 1.78] *p* < 0.001**
Overall Desirability	**-0.27 (-2.86)**	**[-0.46, -0.09] *p* = 0.004**	-0.09 (-0.91)	[-0.27, 0.10] *p* = 0.365
Trial Order	-0.05 (-0.39)	[-0.30, 0.20] *p* = 0.695	0.05 (0.49)	[-0.15, 0.25] *p* = 0.624
Relative Desirability X Trial Order	0.19 (1.41)	[-0.08, 0.47] *p* = 0.158	0.12 (1.15)	[-0.08, 0.32] *p* = 0.248
Overall Desirability X Trial Order	0.11 (1.31)	[-0.06, 0.28] *p* = 0.189	0.01 (0.21)	[-0.12, 0.15] *p* = 0.833
