# Supplementary material for: Rejection-based choices discourage people from opting out of voting
Source: Nat Commun. 2026 Jan 28;17:1768. doi: 10.1038/s41467-026-68472-7 (PMC12916904; doi:10.1038/s41467-026-68472-7)
Supplement: Supplementary file 2 — Reporting Summary [file 41467_2026_68472_MOESM2_ESM.pdf]

nature portfolio

Corresponding author(s): Yi-Hsin Su, Amitai Shenhav

Last updated by author(s): Dec 19, 2025

Reporting Summary

Nature Portfolio wishes to improve the reproducibility of the work that we publish. This form provides structure for consistency and transparency in reporting. For further information on Nature Portfolio policies, see our Editorial Policies and the Editorial Policy Checklist.

Statistics

For all statistical analyses, confirm that the following items are present in the figure legend, table legend, main text, or Methods section.

n/a	Confirmed	
☐	☒	The exact sample size (*n*) for each experimental group/condition, given as a discrete number and unit of measurement
☐	☒	A statement on whether measurements were taken from distinct samples or whether the same sample was measured repeatedly
☐	☒	The statistical test(s) used AND whether they are one- or two-sided<br>*Only common tests should be described solely by name; describe more complex techniques in the Methods section.*
☐	☒	A description of all covariates tested
☐	☒	A description of any assumptions or corrections, such as tests of normality and adjustment for multiple comparisons
☐	☒	A full description of the statistical parameters including central tendency (e.g. means) or other basic estimates (e.g. regression coefficient) AND variation (e.g. standard deviation) or associated estimates of uncertainty (e.g. confidence intervals)
☐	☒	For null hypothesis testing, the test statistic (e.g. *F*, *t*, *r*) with confidence intervals, effect sizes, degrees of freedom and *P* value noted<br>*Give P values as exact values whenever suitable.*
☒	☐	For Bayesian analysis, information on the choice of priors and Markov chain Monte Carlo settings
☒	☐	For hierarchical and complex designs, identification of the appropriate level for tests and full reporting of outcomes
☒	☐	Estimates of effect sizes (e.g. Cohen's *d*, Pearson's *r*), indicating how they were calculated

Our web collection on statistics for biologists contains articles on many of the points above.

Software and code

Policy information about availability of computer code

Data collection	The online experiments (Study 1 and Study 2) were programmed with Psychopy (v2022.2.5). The online survey experiments (Study 3 and Study 4) were set up with Qualtrics.
Data analysis	All statistical and simulation analyses were performed in R (4.4.0), using R packages lme4 (1.1.37), lmerTest (3.1.3), simr (1.0.7) and brunnermunzel (2.0). All analysis scripts are publicly available upon publication, via the GitHub link provided in the manuscript.

For manuscripts utilizing custom algorithms or software that are central to the research but not yet described in published literature, software must be made available to editors and reviewers. We strongly encourage code deposition in a community repository (e.g. GitHub). See the Nature Portfolio guidelines for submitting code & software for further information.

Data

Policy information about availability of data

All manuscripts must include a data availability statement. This statement should provide the following information, where applicable:

- Accession codes, unique identifiers, or web links for publicly available datasets
- A description of any restrictions on data availability
- For clinical datasets or third party data, please ensure that the statement adheres to our policy

All de-identified data, data analysis scripts, and simulation scripts are publicly available at https://doi.org/10.5281/zenodo.17993607.

Research involving human participants, their data, or biological material

Policy information about studies with human participants or human data. See also policy information about sex, gender (identity/presentation), and sexual orientation and race, ethnicity and racism.

Reporting on sex and gender	Information including sex assigned at birth and age was self-reported at the end of the experiments (Studies 1 and 2) or within the online surveys (Studies 3 and 4). We used information about sex and age, alongside with other demographic information, to confirm that our main findings are robust controlling for all of these variables. Our studies include 1,108 females, 992 males, and 16 prefer-not-to-respond participants. Our findings does not only apply to one sex or gender.
Reporting on race, ethnicity, or other socially relevant groupings	Information including party affiliation, race, ethnicity, education levels, parental and maternal education levels was self-reported at the end of the experiments (Studies 1 and 2) or within the online surveys (Studies 3 and 4). We used information about party affiliation, race and ethnicity, education levels, parental and maternal education levels, alongside with sex and age, to confirm that our main findings are robust controlling for all of these variables.
Population characteristics	See above. Also see details for each study below. Across four studies, we did not use representative sampling.<br>Our rationale for the chosen study sample (see Recruitment) is to test general decision mechanisms that underpin decisions to vote, and the role these mechanisms play in decisions when eligible voters are polled about actual political candidates.
Recruitment	All participants were recruited via Prolific. In Studies 1 and 2, we include those who were aged 18-55, fluent in English, living in the States, and have no participation in previous decision-making studies in our lab. In Studies 3 and 4, we were guided by findings in Studies 1 and 2 and added one restriction that only self-identified Independents (filtered by "U.S. Political Affiliation" on Prolific platform) could be included. Since we randomly assigned participants to two experimental conditions and were not investigating individual differences, it is less likely that any self-selection bias have influenced our findings.
Ethics oversight	Studies 1, 2, and 3 were approved by Brown University's Institutional Review Board under protocol 1606001529. Study 4 was approved by UC Berkeley's Committee for the Protection of Human Subjects under protocol 2024-06-17537.

Note that full information on the approval of the study protocol must also be provided in the manuscript.

Field-specific reporting

Please select the one below that is the best fit for your research. If you are not sure, read the appropriate sections before making your selection.

☐ Life sciences ☒ Behavioural & social sciences ☐ Ecological, evolutionary & environmental sciences

For a reference copy of the document with all sections, see nature.com/documents/nr-reporting-summary-flat.pdf

Behavioural & social sciences study design

All studies must disclose on these points even when the disclosure is negative.

Study description	Data was quantitative and experimental. In Studies 1 and 2, participants were instructed to evaluate a series of political policies and make voting decisions between hypothetical candidates. In Studies 3 and 4, participants were instructed to complete an online survey, in which they were polled about their preferences for real-world candidates. The condition of choice was manipulated between-subject.
Research sample	The research samples for all studies consisted of participants from Prolific. Here are the demographic information, each separated to two experimental groups - Selection vs. Rejection:<br>Study 1, Selection: 19 females, 25 males; age = 37.0 (SD = 9.31) ys<br>Study 1, Rejection: 28 females, 19 males; age = 37.3 (SD = 9.79) ys<br>Study 2, Selection: 14 females, 23 males, 2 prefer-not-to-respond; age = 33.9 (SD = 9.30) ys<br>Study 2, Rejection: 25 females, 18 males; age = 33.6 (SD = 9.84) ys<br>Study 3, Selection: 251 females, 230 males, 3 prefer-not-to-respond; age = 35.6 (SD = 9.30) ys<br>Study 3, Rejection: 237 females, 242 males, 4 prefer-not-to-respond; age = 34.9 (SD = 9.30) ys<br>Study 4, Selection: 252 females, 233 males, 4 prefer-not-to-respond; age = 34.6 (SD = 9.38) ys<br>Study 4, Rejection: 282 females, 202 males, 3 prefer-not-to-respond; age = 35.5 (SD = 10.2) ys
Sampling strategy	All studies randomly sampled available participants on Prolific fulfilling our inclusion criteria. For Study 1, in line with the sample sizes of past decision-making studies in our lab (e.g. 30 in Shenhav & Karmakar (2019)), we collected 100 participants for Study 1, aiming at roughly 50 per condition. Confirming that our effect of interest was strong, we collected another 100 participants for Study 2 as a near-replication of Study 1. Both studies ensure sufficient statistical power. For Studies 3 and 4, we based on a pilot study to calculate the sample sizes needed for a two-proportion one-tailed z-test, a power of 0.95, and a type-1 error of 0.05. The minimal sample size for each condition is calculated as 220. Satisfying a power of 0.95, we collected 500 samples for each condition, 1000 samples in total per study. We preregistered both studies (Study 3: https://osf.io/djy4h; Study 4: https://osf.io/89e75) before data collection. Studies 1 and 2 were not preregistered.
Data collection	Online participants performed the study remotely by themselves.
Timing	Data in Study 1 was collected March 8, 2024 - March 10, 2024.

Timing	Data in Study 2 was collected March 10, 2024 - March 12, 2024.<br>Data in Study 3 was collected May 28, 2024.<br>Data in Study 4 was collected September 6, 2024.
Data exclusions	For Studies 1 and 2, we excluded data from those participants who either (1) decided to vote on all ballots without opting out once or (2) had equal to or more than 50% of ballots on which they opted to vote but chose the candidate inconsistent with their assigned goals. Accordingly, we excluded 9 and 18 participants out of 100 in Studies 1 and 2, respectively. For Studies 3 and 4, we preregistered two predictions and their corresponding exclusion criteria: for the first prediction, we excluded 23 and 18 out of 1001 and 1002 collected responses in Studies 3 and 4, respectively, by those who did not claim themselves as eligible voters; for the second prediction, we further excluded 11 and 8 participants in Studies 3 and 4, respectively, by those who responded "Prefer not to respond" in the polling questions.
Non-participation	Participant sign-ups and drop-outs were managed by Prolific and not on our records. All participants who completed the study provided consent, and none declined participation.
Randomization	Participants were randomly assigned to one of the two experimental groups. In Studies 1 and 2, the random assignment relied on a random seed generated by the experimental program PsychoPy; in Studies 3 and 4, the random assignment relied on the Qualtrics built-in function "Randomizer." Across four studies, the investigator who interacted with the study subjects was blinded to the subjects' assigned conditions.

Reporting for specific materials, systems and methods

We require information from authors about some types of materials, experimental systems and methods used in many studies. Here, indicate whether each material, system or method listed is relevant to your study. If you are not sure if a list item applies to your research, read the appropriate section before selecting a response.

Materials & experimental systems

n/a	Involved in the study
☒	☐ Antibodies
☒	☐ Eukaryotic cell lines
☒	☐ Palaeontology and archaeology
☒	☐ Animals and other organisms
☒	☐ Clinical data
☒	☐ Dual use research of concern
☒	☐ Plants

Methods

n/a	Involved in the study
☒	☐ ChIP-seq
☒	☐ Flow cytometry
☒	☐ MRI-based neuroimaging

Plants

Seed stocks	Not involved in our studies
Novel plant genotypes	Not involved in our studies
Authentication	Not involved in our studies
